# Sulforaphane Mitigates Oxidative Stress in Alzheimer's Disease (AD) through NRF2 Activation

**DOI:** 10.1002/alz70861_108046

**Published:** 2025-12-23

**Authors:** Wasi Uzzaman Khan, Abul Kalam Najmi

**Affiliations:** ^1^ Jamia Hamdard, New Delhi, New Delhi India

## Abstract

**Background:**

AD is a neurodegenerative disease that is characterized by inflammation and oxidative stress. A transcription factor called nuclear factor erythroid 2‐related factor 2 (NRF2) controls antioxidant reactions. Sulforaphane (SFN) is a phytochemical that has been shown to activate NRF2 and mitigate oxidative stress.

**Method:**

The present study addresses the impact of SFN on hippocampus NRF2 levels using an animal model of AD produced by Aβ (1–42). Different dosages of SFN were administered to the experimental groups, either with or without combination drugs (trigonilline, TRG; or pioglitazone, PIO). NRF2 levels were evaluated to assess the potential of SFN to modulate oxidative stress.

**Result:**

The study shows that sulforaphane (SFN) administration dose‐dependently recovers NRF2 levels, with 10 mg/kg and 20 mg/kg SFN raising NRF2 levels to roughly 60 pg/mg protein and 80 pg/mg protein compared to Aβ‐exposed groups (30 pg/mg protein), respectively. Significantly, pioglitazone (10 mg/kg) and SFN (10 mg/kg) increased NRF2 levels in a synergistic manner, indicating possible therapeutic advantages. These results underline SFN's potential as a therapeutic drug in Alzheimer's disease models and support the function of NRF2 activation in reducing oxidative stress

**Conclusion:**

These results indicate that SFN is a viable treatment option for reducing oxidative stress in AD, especially when paired with PIO. The importance of combination drugs in strengthening therapeutic efficacy is shown by the synergistic effect of SFN and PIO on NRF2 activation. To fully understand the intricate molecular mechanics and long‐term effectiveness of this strategy, more investigation needs to be conducted.

